# A Single-Longitudinal-Mode S + C Band Wavelength-Tunable Fiber Laser

**DOI:** 10.3390/s24082576

**Published:** 2024-04-17

**Authors:** Da Liu, Yi Jiang

**Affiliations:** 1 School of Optics and Photonics, Beijing Institute of Technology, Beijing 100081, China; liuda@bit.edu.cn; 2 Key Laboratory of Photonic Information Technology, Ministry of Industry and Information Technology, Beijing 100081, China

**Keywords:** fiber laser, external cavity, single mode, fiber sensor demodulation, wavelength-tunable laser

## Abstract

An external cavity wavelength-fiber ring laser (ECWTFL) based on a semiconductor optical amplifier and a combined wavelength scanning filter in the Littrow configuration is proposed and experimentally demonstrated. With the benefit of the combination of an external cavity wavelength filter and a Lyot filter, the laser achieves a single-mode narrow linewidth output with a linewidth of 1.75 kHz. The wavelength tuning range reaches 133 nm, covering the entire S + C band. The proposed ECWTFL is used for demodulation of a fiber EFPI sensor; the result shows that the proposed ECWTFL has the ability to demodulate the small cavity-length FPI sensor.

## 1. Introduction

A wavelength-tunable fiber laser (WSTL) is a powerful tool for many applications such as optical fiber sensing [1], optical communication [2], and spectroscopy [3]. As the development of WSTLs has had a significant impact in terms of promoting progress in these fields, researchers in related industries are investing energy into research on WSFLs. At present, research on WSTLs mainly focuses on the gain medium, the configuration of the lasers, and the wavelength scanning filter, in order to achieve narrow linewidth, single longitudinal mode, high-speed scanning, and wide wavelength range. The most widely used gain media for WSTLs are semiconductor optical amplifiers (SOAs) [4,5,6,7,8] and rare-earth-doped fibers [9,10,11,12], while the configuration of a WSTL is often based on a line cavity [10,13] or loop cavity [11,14,15]. There are many types of wavelength scanning filters for WSTLs, such as configurations based on fiber Bragg gratings (FBGs) [10,16], fiber Fabry-Pérot-tunable filters (FFP-TFs) [8,17,18], electro-optic/acousto-optic modulators (EOMs/AOMs) [4], optical fiber birefringence [9,15], and a diffraction grating combined with a polygon scanner mirror (PSM) [3,5,13,19].

An external cavity wavelength-tunable fiber laser (ECWTFL) has the advantages of a narrow linewidth, a wide wavelength scanning range, and a flexible structure [20,21,22]. In particular, an ECWTFL with a wavelength scanning filter based on a diffraction grating and PSM has the characteristics of ultrahigh wavelength scanning speed, customizability, and wavelength unidirectional scanning [23]; in view of these advantages, it is favored by researchers in several fields of application, and has been developed intensively over the last two decades. However, studies of wavelength scanning filters with a configuration based on a combination of a diffraction grating and PSM have almost all involved wavelength-swept lasers in the 1.3 μm band, and there have been very few studies in a wavelength band of 1.5 μm, which is the operational band for optical fiber sensing, such as optical frequency domain reflectometry (OFDR) [24,25,26,27], white-light interferometry technology [28], and demodulation of fiber optic Fabry-Pérot interferometric (FPI) sensors [29,30]. Hence, research on high-speed, wide-wavelength-range WSTLs in the 1.5 μm wavelength band has important value.

This paper presents an ECWTFL that uses a Lyot filter and a blazed grating for wavelength selection. A PSM was combined with a diffraction grating to create the wavelength scanning filter and external cavity. An SOA was used as the gain medium, and an optical polarization-maintaining isolator (OPMI) with panda-style pigtails was applied to realize the unidirectional transmission of light in the ring loop. A Lyot filter was formed by a polarization controller (PC) and the OPMI, which could suppress the mode hopping and achieve a narrow linewidth output of the laser. Our experimental results show that the proposed ECWTFL operates in single-longitudinal-mode (SLM) with a linewidth of 1.75 kHz; the wavelength tuning range is about 1452–1586 nm, and covers the entire S + C band.

## 2. Experimental Setup

**Figure 1:**
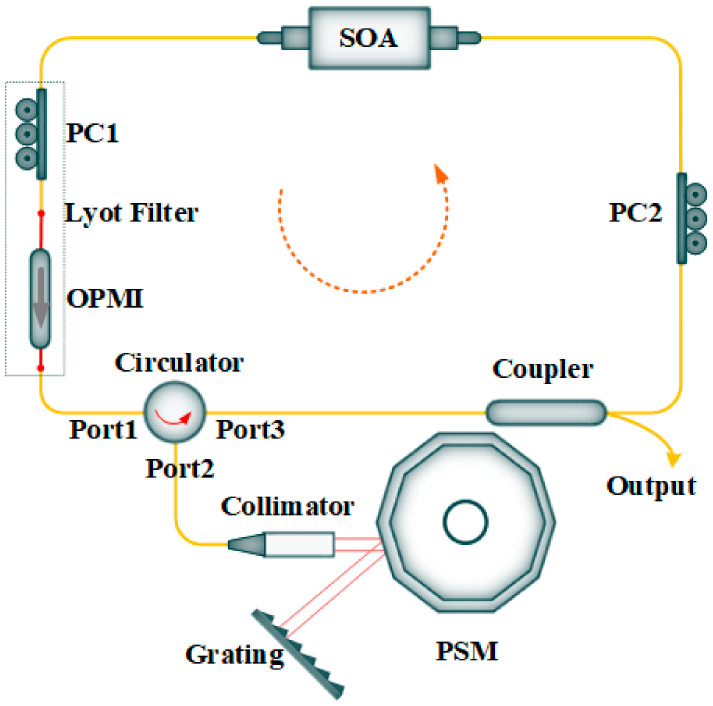
Experimental setup of the ring loop external cavity wavelength-tunable fiber laser (SOA: semiconductor optical amplifier, OPMI: optical polarization-maintaining isolator, PC: polarization controller, red points: fusion points between PMF and SMF, PSM: polygon scanner mirror).

The experimental setup of the proposed ECWTFL is shown in Figure 1. The ECWTFL was combined with an optical fiber ring loop and an external cavity. The optical fiber ring loop included a commercial SOA (IPSAD1501, INPHENIX, Livermore, California, USA), an OPMI, two polarization controllers (PC1 and PC2), an optical circulator, and a 1 × 2 optical coupler with a splitting ratio of 80:20.

The external cavity, which also worked as a wavelength selection configuration, comprised an aspheric collimator (GCX-LF6APC-1550, DHC, Beijing, China) with a focusing length of 6.38 mm, a PSM (SA24C, Lincoln Laser, Ontario, Canada) with 10 facets and a maximum rotating speed of 24,000 RPM, and a diffraction grating (GR25-0616, Thorlabs, Newton, New Jersey, USA) with 600 lines/mm and a blaze angle of 28°48′. All the optical fiber components used in the experimental setup had single-mode optical fiber (SMF) pigtails, except the OPMI, which had two panda-style polarization-maintaining optical fiber (PMF) pigtails. The OPMI with PMF pigtails and PC1 formed a Lyot filter [31].

One end of the SOA was connected to PC1, which fusion-spliced with the input of the OPMI, and the other end was connected to the PC2. The combination of two PCs and the OPMI can induce polarization-dependent loss to suppress the mode competition and mode hopping. The output of the OPMI was fusion-spliced with Port 1 of the optical circulator. The PC1 and OPMI could realize the unidirectional transmission and single polarization mode operation of light in the ring loop. Port 2 of the optical circulator was connected to the aspheric collimator, which was coated with broadband antireflective film and was used to collimate the light incident on the facet mirror of the polygon scanner. The light was reflected out by the PSM and was incident on the diffraction grating at approximately the blaze angle. The first order of diffraction light was fed back along the original optical path, passed through the aspheric collimator, injected into Port 2 and passed through Port 3 of the optical circulator, and then returned to the optical fiber ring loop. 

The main optical fiber ring loop length was about 12 m, the optical fiber length from Port 2 (circulator) to the collimator was about 2 m, and the external air cavity length was about 0.25 m, so the total length of the ECWTFL was about 23.86 m (12 × 1.468 + 2 × 2 × 1.468 + 0.25 × 2 m), and the corresponding longitudinal mode spacing was about 12.51 MHz.

Wavelength scanning was achieved by rotating the PSM to change the angle of incidence of the light on the diffraction grating. Port 3 of the optical circulator was connected to the single port of the 1 × 2 optical coupler. Of the two ports at the other end of the optical coupler, the 80% port was connected to the PC2, while the 20% port operated as the laser output.

## 3. Results and Discussion

**Figure 2:**
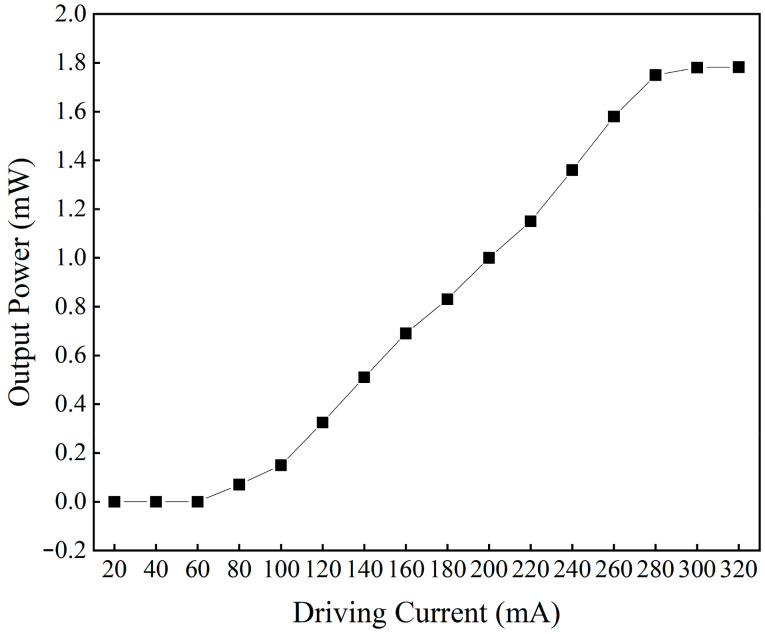
The output power of the ECWTFL with different driving currents of the SOA.

The laser output of SOA at different driving currents was tested, and the output results are shown in Figure 2. As can be seen, the threshold current of SOA is 60 mA, and the gain saturation current is 280 mA. There have been many studies on SOA-based wavelength-swept lasers with different driving currents [32,33,34], and this paper did not do further research. In all our experiments, the driving current of the SOA was set to 240 mA.

### 3.1. Wavelength Output Characteristics

**Figure 3:**
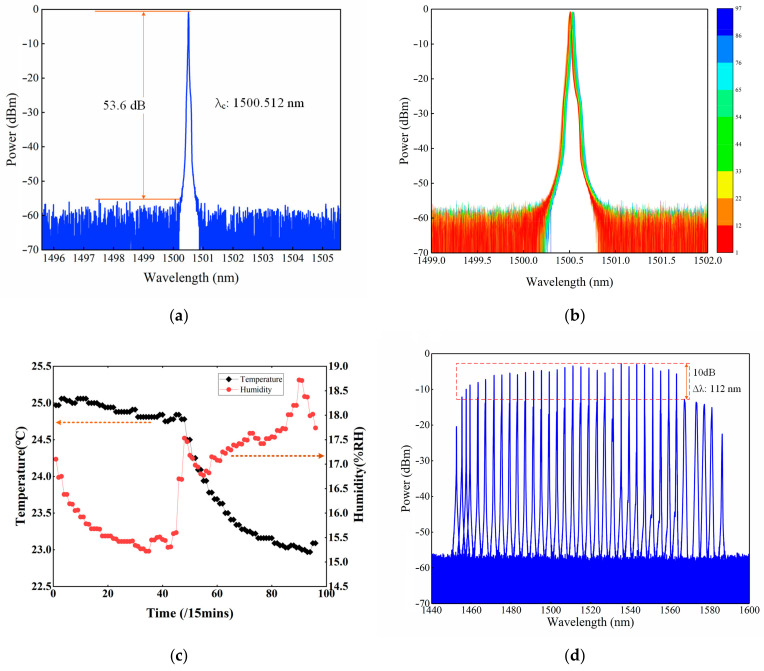
(**a**) The spectrum of single wavelength operation; (**b**) Spectrum stability of the ECWTFL output; (**c**) Changes in environmental temperature and relative humidity; (**d**) Wavelength tuning range of the proposed ECWTFL.

By tuning the angle of the PSM and diffraction grating, and then tuning the two PCs carefully, the proposed ECWTFL was operated in a single wavelength output. The output spectrum at a center wavelength of 1500.512 nm was tested using an optical spectrum analyzer (OSA, AQ6370D, YOKOGAWA, Tokyo, Japan) with a resolution of 0.02 nm, and the result is shown in Figure 3a. The optical signal to noise ratio (OSNR) is about 53.6 dB. 

The stability of the proposed ECWTFL output with single wavelength operation was tested using the OSA (wavelength scanning span: 3 nm, resolution: 0.02 nm, wavelength sampling interval: 0.004 nm). The OSA was programmed to save the spectra automatically in the external storage every 15 min. The spectra were recorded over 24 h, and the results are shown in Figure 3b. At the same time, the environmental temperature and relative humidity were also recorded, as shown in Figure 3c. It can be seen from Figure 3c that the temperature decreased by about 2 °C while the relative humidity changed by 3.5%RH, corresponding to a wavelength red shift of <0.03 nm and a power decrease of about 0.5 dBm for the ECWTFL output. This indicates that the presented ECWTFL is influenced by the environmental conditions, but also exhibits high stability over dozens of hours.

The wavelength tuning range of the proposed ECWTFL was tested using OSA, for which the wavelength scanning range was set from 1440 to 1600 nm, and the wavelength resolution and sampling interval were set to 0.1 nm and 0.02 nm, respectively. By precisely rotating the angle of the PSM each time, a wavelength interval of about 3 nm for the ECWTFL output was achieved, and the spectrum was saved in the internal storage of the OSA. By repeating the above operation, all the spectra for the full wavelength tuning range were obtained, as shown in Figure 3d. It can be observed that the full wavelength tuning range of the ECWTFL exceeds 133 nm from 1452.56 nm to 1586.34 nm, and the 10 dB bandwidth exceeds 112 nm from 1455.3 nm to 1567.32 nm.

### 3.2. Linewidth Measurement

**Figure 4:**
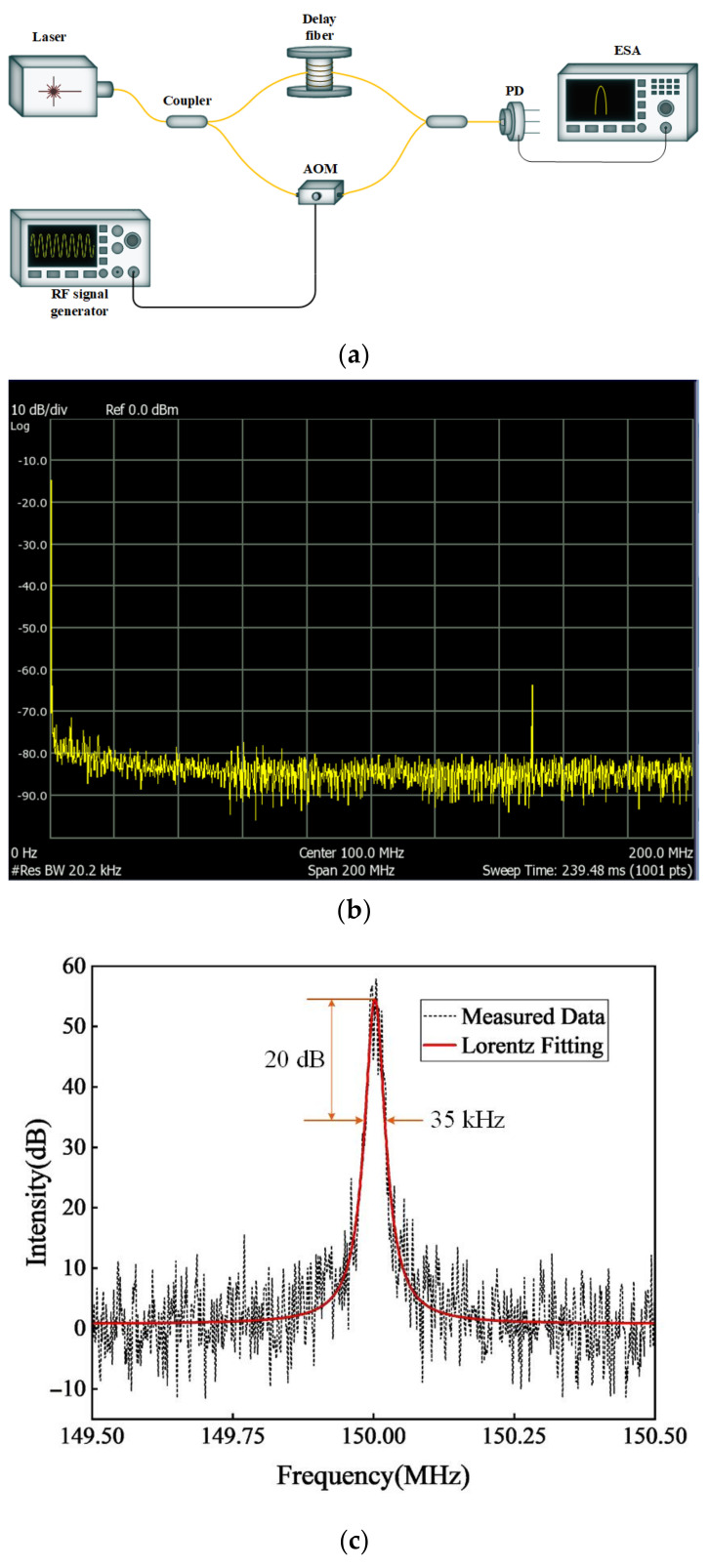
(**a**) Linewidth experimental setup with the delayed self-homodyne method (AOM: acoustic optical modulator, ESA: electrical spectrum analyzer); (**b**) Beating signal measured by ESA in the frequency range of 0–200 MHz; (**c**) Beating signal with a Lorentz fitting.

The linewidth of the ECWTFL at 1550 nm was measured using the delayed self-homodyne method with an optical fiber Mach–Zehnder interferometer (MZI). The experimental setup is shown in Figure 4a. The delay optical fiber of one arm of the MZI was set to 60 km, while the other arm of the MZI generated a frequency shift of about 150 MHz by using an acoustic optical modulator (AOM, SGTF150-1550-1S, CETC, Chongqing, China) with an RF signal generator (AFG3252C, Tektronix, Oregon, USA). The output of the ECWTFL was connected to the input of the MZI, and the self-homodyne signal was detected by the photodetector (PD) and electrical spectrum analyzer (ESA, N1996A, Agilent Technologies, CA, USA). As shown in Figure 4b, there is only one strong beating signal in the frequency range of 0–200 MHz, which means the ECWTFL is operating in single-longitudinal-mode. In order to measure the linewidth of the ECWTFL precisely, the bandwidth jof the ESA was set to 149.5–150.5 MHz with a BW resolution of 1 kHz, and a bandwidth of 35 kHz at −20 dB was achieved with a Lorentz line shape fitting; the beating signal is shown in Figure 4c. Due to the measured laser linewidth being considered as 1/20 of the −20 dB Lorentz line bandwidth [35], the linewidth of the ECWTFL is 1.75 kHz.

### 3.3. Using FPI Sensor Demodulation

**Figure 5:**
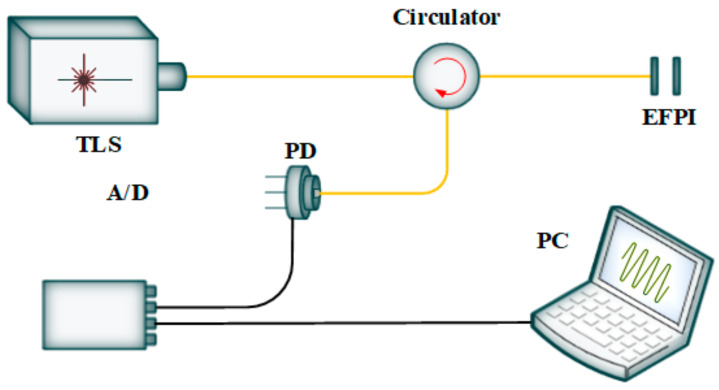
The experimental setup of the EFPI sensor demodulation.

**Figure 6:**
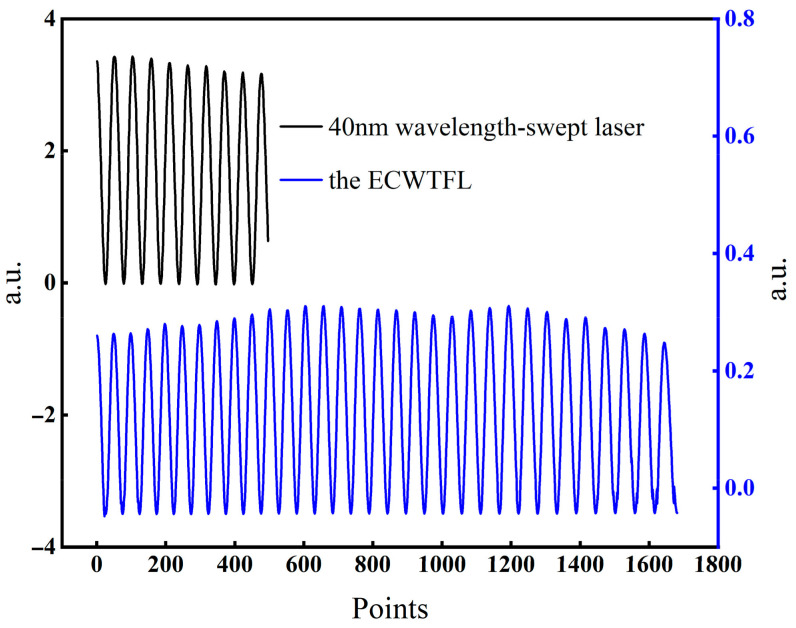
The interference spectra of the two lasers.

In order to test the demodulation performance of the proposed ECWSTL for the fiber FPI sensors, a test setup was constructed as shown in Figure 5. The wavelength-tunable laser light source (TLS) was injected through Port 1 of a circulator into an external cavity FPI sensor (EFPI), which was connected to Port 2. The cavity length of the EFPI could be adjusted by a micrometer. The interference spectrum of the EFPI entered the PD through Port 3, and the PD converted the optical signal into an electrical signal, and then transmitted it to a computer through an A/D card for data display and analysis. TLS uses a commercial 40 nm wavelength-swept laser and the proposed ECWSTL, respectively. The acquired interference spectra are shown in Figure 6 when the EFPI cavity was adjusted to a length. As can be seen from the results, the EFPI interference spectrum obtained when using ECWSTL as the light source was about three times that of using the commercial wavelength-swept laser, which matches the wavelength range of the ECWSTL previously tested. The experimental results indicate that the proposed ECWSTL can be used to demodulate FPI sensors with small cavity lengths.

The cavity length of an EFPI can be calculated by Equations (1) and (2) [36]. It is easy to calculate by Equation (1); when the cavity length of the EFPI is about 30 μm, the corresponding interference spectrum peak to peak wavelength interval is about 40 nm. At this time, when the commercial 40 nm wavelength-swept laser is used as the light source of a demodulator, the cavity length of an EFPI can no longer be demodulated. However, by using the proposed ECWTFL, 2–3 complete interference peaks can be scanned, and the cavity length of the EFPI can be demodulated.

d = λ_1_λ_2_/2Δλ, (1)

Δλ = |λ_1_ − λ_2_|, (2)

where d is the cavity length of an EFPI, λ_1_ and λ_2_ are the wavelengths of two adjacent interference peaks in the interference spectrum of the EFPI, respectively, and Δλ is the difference between λ_1_ and λ_2_.

**Figure 7:**
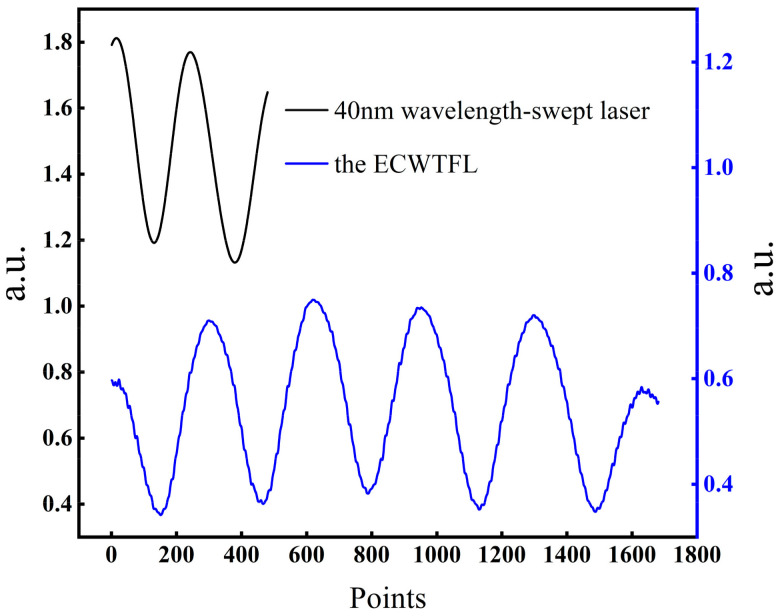
The interference spectra of the two lasers with an EFPI cavity length of about 50 μm.

By adjusting the knob of the micrometer, the cavity length of the EFPI is adjusted to about 50 µm. Then, the interference spectrum of the EFPI is tested by using the experimental setup in Figure 5. The commercial 40 nm wavelength-swept laser and the proposed ECWTFL are used as the scanning light source, respectively; the interference spectra obtained are shown in Figure 7. It can be seen from the diagram that when using the 40 nm wavelength-swept laser, only one effective interference peak can be obtained, and the cavity length of the EFPI can no longer be calculated by using Equations (1) and (2). On the other hand, four interference peaks can be obtained by using the ECWTFL as the scanning light source, which can effectively demodulate the cavity length of an EFPI by using Equation (1).

## 4. Conclusions

This paper has presented and experimentally demonstrated a 1.5 μm wideband ring loop ECWTFL. An SOA operated as the laser amplifier. An external cavity based on a Littrow configuration and a Lyot filter, which is formed by a PC and OPMI, is used as the wavelength selector. Our experimental results indicate that the ECWTFL wavelength tuning range covers the entire S + C band from 1452.56 nm to 1586.34 nm, the 10 dB wavelength is about 112 nm, and the linewidth is 1.75 kHz at 1550 nm. The ECWTFL also exhibits good stability over 24 h. The performance of the ECWTFL for demodulation of fiber optic FPI sensors was tested, and the results show that this laser can be used as a light source for the demodulation of small cavity-length FPI sensors.

## Data Availability

Data are contained within the article.
